# Beyond Stenosis: Mechanism-Based Multimodality Imaging and Invasive Coronary Function Testing for Endotype Definition in ANOCA/INOCA

**DOI:** 10.3390/medicina62050910

**Published:** 2026-05-08

**Authors:** Lucio Giuseppe Granata, Marcello Marchetta, Simona Giubilato, Giuseppe Massimo Sangiorgi, Giuseppina Maura Francese, Giuseppe Andò

**Affiliations:** 1 Cardiology Division, Garibaldi-Nesima Hospital, ARNAS Garibaldi, 95122 Catania, Italy; 2 Cardiology Department, Policlinico Tor Vergata, 00133 Rome, Italy; marcello.marchetta1997@gmail.com (M.M.); g.sangiorgi@gmail.com (G.M.S.); 3 Cardiology Division, Cannizzaro Hospital, 95126 Catania, Italy; simogiub@hotmail.com; 4 Cardiology Department, Azienda Ospedaliera Papardo, 98158 Messina, Italy; giuseppeando1975@gmail.com; 5 Department of Clinical and Experimental Medicine, University of Messina, 98122 Messina, Italy

**Keywords:** ANOCA, INOCA, coronary microvascular dysfunction, coronary vasospasm, myocardial blood flow, coronary flow reserve, invasive coronary function testing, multimodality imaging, PET, CMR

## Abstract

Angina or objective myocardial ischaemia in the absence of obstructive coronary artery disease, referred to as ANOCA/INOCA, represents a prevalent and clinically significant condition associated with persistent symptoms, impaired quality of life, and increased healthcare utilisation. Contemporary evidence has reframed these syndromes as manifestations of coronary vascular dysfunction, encompassing structural and functional coronary microvascular dysfunction, epicardial vasospasm, microvascular spasm, and mixed phenotypes. In this context, multimodality imaging should not be conceptualised as sequential test accumulation, but rather as a structured, mechanism-based diagnostic strategy aimed at defining the underlying coronary endotype. The 2024 ESC Guidelines for chronic coronary syndromes endorse dedicated diagnostic pathways beyond a stenosis-centred paradigm and support the use of invasive coronary function testing (ICFT) in selected patients with persistent symptoms or inconclusive non-invasive findings. An integrated approach combining anatomical assessment (coronary computed tomography angiography or invasive angiography ± pressure-based indices), quantitative perfusion imaging (positron emission tomography or stress cardiovascular magnetic resonance), and ICFT (including coronary flow reserve, microvascular resistance indices, and acetylcholine provocation testing) enables comprehensive characterisation of coronary physiology and vasomotor function. This review proposes a pragmatic framework linking diagnostic findings to targeted therapy through a test-to-endotype-to-therapy paradigm. We summarise the strengths and limitations of each modality, discuss implementation challenges, and highlight the clinical relevance of endotype-driven management. By shifting from a stenosis-centred to a physiology- and mechanism-based approach, this strategy has the potential to close the longstanding gap between diagnosis and treatment in patients with ischaemia beyond obstructive coronary disease.

## 1. Introduction

A substantial proportion of patients undergoing evaluation for chronic chest pain or suspected myocardial ischaemia are found to have non-obstructive coronary arteries at invasive coronary angiography (ICA) or coronary computed tomography angiography (CCTA). Historically, these patients were frequently reassured or labelled as having non-cardiac symptoms, reflecting a diagnostic paradigm largely centred on the presence or absence of flow-limiting epicardial stenosis. However, this approach fails to account for the complexity of myocardial ischaemia and overlooks a substantial burden of coronary vascular dysfunction [1,2].

Emerging evidence across coronary syndromes also demonstrates that clinically relevant events frequently arise from non-obstructive lesions driven by biological activity rather than luminal stenosis alone [3].

Contemporary evidence has fundamentally redefined angina or ischaemia with non-obstructive coronary arteries (ANOCA/INOCA) as a clinically meaningful syndrome rather than a diagnosis of exclusion. In many patients, symptoms are driven by abnormalities of coronary vascular function, including structural and functional coronary microvascular dysfunction, epicardial vasospasm, microvascular spasm, or mixed endotypes [2,4]. Standardised diagnostic criteria from the Coronary Vasomotion Disorders International Study Group (COVADIS) have provided a reproducible framework for defining vasospastic angina and microvascular angina, enabling more consistent phenotyping across studies and clinical practice [5,6].

Importantly, ischaemic findings in this population, historically considered “false positive” in the absence of obstructive coronary disease, may instead reflect true coronary microvascular or vasomotor dysfunction. In a contemporary invasive physiology study, abnormal exercise electrocardiographic findings in patients with ANOCA were closely associated with coronary microvascular dysfunction, challenging the traditional assumption that the absence of obstructive epicardial disease implies a non-ischaemic mechanism [7].

The 2024 ESC Guidelines for chronic coronary syndromes formally integrate ANOCA/INOCA within the CCS framework and support structured diagnostic pathways extending beyond anatomical imaging alone [1]. Within this paradigm, the central clinical question is no longer limited to whether ischaemia is present, but rather which underlying mechanism is responsible and how this should guide treatment. This shift reflects a broader transition from anatomy-based to mechanism-based cardiovascular medicine, with increasing emphasis on comprehensive functional assessment of the coronary circulation [8].

A major limitation of traditional approaches is the reliance on anatomical assessment alone. The severity of epicardial stenosis does not reliably predict its physiological significance, and pressure-based indices such as fractional flow reserve (FFR), while essential for evaluating obstructive disease, do not fully capture coronary flow dynamics. Discordance between pressure-based and flow-based indices is common and reflects the independent contribution of the coronary microcirculation to myocardial perfusion [9]. Consequently, patients may exhibit persistent symptoms and objective ischaemia despite non-obstructive coronary arteries or non-significant pressure gradients.

In this context, multimodality imaging requires redefinition. Rather than representing sequential test accumulation, it should be understood as a structured, endotype-oriented diagnostic strategy. A pragmatic approach integrates: (i) anatomical assessment to exclude obstructive coronary artery disease and define plaque burden, (ii) quantitative perfusion imaging to detect global or regional flow impairment and (iii) invasive coronary function testing (ICFT) to define coronary endotypes and guide stratified therapy [1,2,4,8].

This review aims to provide a clinically actionable and ESC-aligned synthesis of multimodality imaging and invasive coronary function testing in ANOCA/INOCA. We propose a mechanism-based framework linking diagnostic findings to pathophysiological endotypes and targeted treatment, with the goal of transitioning from descriptive diagnosis to precision cardiovascular care.

## 2. Materials and Methods

This narrative review was conducted through a structured, non-systematic literature search aimed at identifying clinically relevant evidence on the pathophysiology, diagnosis, and management of ANOCA/INOCA, with particular emphasis on multimodality imaging and invasive coronary function testing.

Electronic searches were performed in PubMed, Embase, and Web of Science for articles published up to January 2026. Search terms included combinations of “ANOCA”, “INOCA”, “coronary microvascular dysfunction”, “microvascular angina”, “vasospastic angina”, “microvascular spasm”, “coronary vasomotor disorders”, “myocardial blood flow”, “myocardial flow reserve”, “positron emission tomography”, “stress cardiovascular magnetic resonance”, “coronary flow reserve”, “index of microcirculatory resistance”, “acetylcholine testing”, and “invasive coronary function testing”.

Eligible sources included contemporary international guidelines, expert consensus documents, state-of-the-art reviews, randomised and prospective clinical studies, multicentre registries, and clinically relevant observational studies addressing diagnostic or therapeutic strategies in ANOCA/INOCA. Priority was given to evidence aligned with contemporary ESC recommendations, studies providing mechanistic or prognostic insight, and publications directly informing endotype-based diagnosis or management.

Articles focused exclusively on obstructive coronary artery disease, acute coronary syndromes without relevance to non-obstructive coronary syndromes, non-coronary causes of chest pain, or purely preclinical mechanisms without clinical applicability were not prioritised. Reference lists of key documents were manually screened to identify additional relevant publications.

Given the narrative nature of the review, no formal protocol registration, systematic risk-of-bias assessment, or quantitative synthesis was performed. Therefore, the selection and interpretation of evidence may be subject to narrative selection bias. To mitigate this limitation, the manuscript prioritised guideline documents, consensus statements, pivotal trials, prospective registries, and reproducible diagnostic criteria, while explicitly framing the proposed pathway as a clinically pragmatic synthesis rather than a systematic evidence hierarchy.

## 3. Definitions and Clinically Actionable Endotypes

ANOCA/INOCA comprises multiple coronary endotypes with distinct diagnostic and therapeutic implications. A pragmatic classification into four principal endotypes is clinically useful, while acknowledging frequent overlap in real-world practice [1,2,4].

### 3.1. Structural Coronary Microvascular Dysfunction (CMD)

This endotype is characterised by impaired vasodilatory capacity and/or increased minimal microvascular resistance, reflecting structural alterations of the coronary microcirculation, including rarefaction, remodelling and perivascular fibrosis. It is typically identified by reduced myocardial flow reserve (MFR) on quantitative perfusion imaging, positron emission tomography (PET) or stress cardiovascular magnetic resonance (CMR), or by reduced coronary flow reserve (CFR) in association with elevated microvascular resistance indices, such as the index of microcirculatory resistance (IMR), during invasive coronary function testing [5,6,8].

### 3.2. Functional/Endothelial CMD

Functional CMD is characterised by impaired endothelial-mediated vasodilation and abnormal regulation of vascular tone. In contrast to structural CMD, microvascular resistance may be normal or only mildly elevated, while abnormalities are primarily related to endothelial dysfunction and impaired nitric oxide bioavailability. This phenotype may present with reduced CFR and/or abnormal vasoreactivity during invasive testing and frequently overlaps with vasospastic disorders [2,6].

### 3.3. Epicardial Vasospasm (Vasospastic Angina)

Epicardial vasospasm is defined by transient, reversible constriction of epicardial coronary arteries leading to myocardial ischaemia. According to COVADIS criteria, diagnosis requires the reproduction of symptoms with ischaemic electrocardiographic changes and angiographic evidence of ≥90% vasoconstriction during provocative testing, typically with intracoronary acetylcholine [4]. This endotype reflects hyperreactivity of vascular smooth muscle and endothelial dysfunction and represents a dynamic disorder of coronary tone regulation and is increasingly recognised in contemporary clinical practice [10].

### 3.4. Microvascular Spasm

Microvascular spasm is characterised by angina and ischaemic electrocardiographic changes during acetylcholine provocation in the absence of significant epicardial constriction. This phenotype reflects abnormal vasomotor reactivity (hyperreactivity) at the level of the microcirculation and cannot be detected without dedicated functional testing and is therefore frequently underdiagnosed [4,8].

Importantly, all these endotypes are not mutually exclusive. Mixed phenotypes, particularly the coexistence of CMD and vasospasm, are common and clinically relevant. Contemporary registry data suggest that a large proportion of ANOCA patients exhibit overlapping mechanisms, highlighting the need for comprehensive functional evaluation rather than binary classification [6,11]. This reinforces the concept that endotyping should be viewed as a spectrum of interacting mechanisms rather than discrete categories.

Emerging data further suggest that the distribution of endotypes may be influenced by biological factors such as sex, with microvascular dysfunction more frequently observed in women and epicardial vasomotor disorders more prevalent in men [12]. These findings highlight the need for comprehensive physiological assessment rather than reliance on single-modality testing.

Overall, this framework reinforces that ANOCA/INOCA should be approached as a spectrum of coronary vascular dysfunction. Accurate endotype definition requires integration of anatomical imaging, quantitative perfusion assessment, invasive coronary function testing, and represents the cornerstone of mechanism-based management.

## 4. Pathophysiological Rationale

Coronary vascular dysfunction represents the main pathophysiological substrate underlying ANOCA/INOCA and includes a heterogeneous spectrum of abnormalities involving both the epicardial coronary arteries and the coronary microcirculation [13,14]. Despite their mechanistic diversity, these mechanisms ultimately converge on a final common pathway of inadequate myocardial blood flow relative to metabolic demand, resulting in myocardial ischaemia [15].

### 4.1. Coronary Flow as the Final Common Pathway

A fundamental principle of coronary physiology is that myocardial oxygen extraction at rest is already high and only modestly augmentable; accordingly, increases in myocardial oxygen demand are met predominantly through proportional increases in coronary blood flow rather than further oxygen extraction [16,17]. Consequently, the capacity of the coronary circulation to augment flow, rather than the mere presence of anatomical stenosis, is the key determinant of perfusion adequacy [16,18]. This concept highlights a critical limitation of stenosis-centred paradigms: epicardial coronary stenosis represents only one of several possible determinants of impaired flow and its anatomical severity does not reliably predict its physiological impact [18,19,20,21]. Conversely, even in the absence of obstructive epicardial disease, impaired coronary flow reserve may arise from microvascular dysfunction, vasospastic disorders, or both [4,13].

### 4.2. Pressure-Based Versus Flow-Based Physiology

The distinction between pressure-based and flow-based indices is central to understanding ANOCA/INOCA. FFR quantifies pressure gradients across epicardial stenoses and remains the reference standard for defining the haemodynamic significance of obstructive coronary artery disease [14,19,21]. However, as a pressure-derived index, FFR does not directly measure coronary blood flow and therefore does not account for abnormalities related to non-focal atherosclerotic disease or the coronary microcirculation, both of which are key determinants of ischaemia in ANOCA/INOCA [2,18,20,22].

In contrast, flow-based indices such as CFR and MFR reflect the integrated capacity of the coronary circulation to augment blood flow, capturing the combined contribution of epicardial and microvascular compartments [4]. Accordingly, patients with preserved FFR but reduced CFR or MFR represent a distinct physiological phenotype, typically associated with coronary microvascular dysfunction and/or generalised atherosclerotic burden, and characterised by persistent symptoms and worse clinical outcomes despite the absence of focal flow-limiting stenosis [23,24,25,26].

### 4.3. Mechanisms of Coronary Microvascular Dysfunction

CMD encompasses a spectrum of structural and functional abnormalities affecting vessels below the resolution of conventional angiography. Structural CMD is characterised by microvascular remodelling, luminal narrowing, rarefaction, and perivascular fibrosis, leading to increased minimal microvascular resistance and impaired vasodilatory capacity [27].

Functional CMD, in contrast, is primarily driven by endothelial dysfunction and impaired nitric oxide bioavailability, resulting in abnormal regulation of vascular tone and attenuated vasodilatory responses. These abnormalities may be exacerbated by inflammation, oxidative stress, and cardiometabolic comorbidities, including diabetes, obesity, and hypertension [28].

### 4.4. Vasomotor Disorders and Dynamic Flow Limitation

In contrast to CMD, vasospastic disorders are characterised by dynamic, reversible reductions in coronary blood flow due to transient vasoconstriction. These abnormalities may involve the epicardial arteries or the microcirculation and are related to a complex interplay between endothelial dysfunction and vascular smooth muscle cell hyperreactivity [29,30,31,32]. The interaction between impaired vasodilation (CMD) and enhanced vasoconstriction (vasospasm) contributes to the heterogeneity of clinical presentation and may explain variability in symptoms, test results, and treatment response [4,13,33].

### 4.5. Prognostic Implications of Flow Impairment

A key advance in the field has been the recognition that coronary vascular dysfunction is not a benign condition. Impaired coronary flow reserve, assessed either invasively or by quantitative imaging, has consistently been associated with an increased risk of major adverse cardiovascular events, independent of the presence of obstructive coronary artery disease. Large cohort studies using PET have demonstrated that reduced myocardial flow reserve is a strong and independent predictor of mortality and cardiovascular events, even in patients with angiographically normal or non-obstructive coronary arteries [34,35].

Similarly, invasive physiological studies have shown that abnormalities in CFR and microvascular resistance indices are associated with adverse outcomes and persistent symptom burden [36].

These findings reinforce that coronary vascular dysfunction is a pathophysiologically relevant and prognostically important disease process rather than a benign or merely functional condition. Moreover, they provide the mechanistic rationale for a shift towards physiology-guided, endotype-based diagnostic and therapeutic strategies [2,4,25,37,38].

### 4.6. From Pathophysiology to Mechanism-Based Care

The clinical relevance of these mechanisms lies in their therapeutic implications. Vasospastic angina is predominantly mediated by abnormal coronary vasoconstriction and typically responds to vasodilator therapy, particularly calcium-channel blockers and nitrates, whereas structural coronary microvascular dysfunction reflects impaired vasodilatory capacity and increased microvascular resistance, often in the context of cardiometabolic risk, and therefore requires a broader risk factor–modifying and microvascular-directed approach [13,14,39,40,41].

This concept is supported by interventional evidence demonstrating that a diagnostic strategy incorporating invasive coronary function testing, followed by stratified therapy based on identified endotypes, leads to significant improvements in angina and quality of life [42,43].

Together, these observations establish coronary vascular dysfunction as a central determinant of myocardial ischaemia in ANOCA/INOCA and provide the pathophysiological foundation for a mechanism-based diagnostic and therapeutic paradigm.

## 5. Non-Invasive Testing: From Ischaemia Detection to Functional Endotyping

Non-invasive imaging plays a central role in the evaluation of ANOCA/INOCA, but its value extends beyond the documentation of inducible ischaemia [1,4]. In this setting, a major objective is the quantitative assessment of coronary flow and flow reserve, with the aim of identifying the underlying functional endotype rather than merely demonstrating regional perfusion defects [2,38,44].

### 5.1. CCTA: An Anatomic Gatekeeper and Plaque Phenotype Tool

CCTA provides a robust non-invasive assessment of coronary anatomy, enabling reliable exclusion of obstructive coronary artery disease and characterisation of plaque burden and morphology [45,46].

Its primary role is to define the presence or absence of epicardial disease and to identify an atherosclerotic substrate that may warrant preventive therapy, even in the absence of flow-limiting stenosis [1,4]. Beyond stenosis assessment, CCTA provides incremental value through plaque characterisation, allowing identification of high-risk features such as low-attenuation plaque, positive remodelling, and spotty calcification, which are associated with adverse cardiovascular outcomes and may influence preventive strategies [1,3,47].

Importantly, a normal or non-obstructive CCTA does not exclude coronary vascular dysfunction. Rather, it increases the likelihood that symptoms arise from microvascular or vasomotor abnormalities, thereby strengthening the indication for downstream functional testing [4]. This distinction is critical to avoid misclassification and inappropriate reassurance in symptomatic patients.

In ANOCA/INOCA, the role of CCTA remains adjunctive, as it is fundamentally an anatomical modality and does not directly assess coronary microvascular dysfunction or vasomotor abnormalities. It cannot replace dedicated functional or invasive endotype-oriented testing. Although primarily anatomical, emerging techniques such as CT-derived fractional flow reserve (FFR-CT) provide a non-invasive estimation of lesion-specific haemodynamic significance. However, in ANOCA/INOCA, where diffuse disease and microvascular dysfunction predominate, FFR-CT has limited ability to capture the full spectrum of coronary vascular dysfunction and should be interpreted within a broader physiological framework [9,22].

### 5.2. PET: Absolute Myocardial Blood Flow and Flow Reserve

PET is the most established non-invasive modality for quantitative assessment of myocardial blood flow (MBF) and MFR [48,49]. Unlike relative perfusion imaging techniques, PET enables absolute measurement of MBF (mL/min/g) at rest and during pharmacological stress, allowing detection of both regional and global impairments in coronary perfusion that may be missed by conventional approaches [50,51].

From a physiological perspective, MFR is defined as the ratio of stress to resting MBF and provides an integrated index of the vasodilatory capacity of the entire coronary circulation [52]. In clinical practice, MFR < 2.0 is generally considered abnormal, with values < 1.5 identifying higher-risk phenotypes. Importantly, reduced MFR is not specific for a single mechanism and may result from [23,53,54]:

• epicardial flow-limiting disease,
• diffuse atherosclerosis,
• structural or functional coronary microvascular dysfunction, or combinations thereof

#### 5.2.1. Diagnostic Patterns and Endotype Inference

The interpretation of PET findings in ANOCA/INOCA relies not only on absolute values but also on flow patterns [49,52]:

A global reduction in MFR is suggestive of CMD, although extensive epicardial atherosclerosis or increased resting flow may contribute [48,49].

Regional reductions in stress MBF or MFR, particularly when aligned with a vascular territory, raise suspicion for focal epicardial disease or mixed physiology [55].

Normal relative perfusion with reduced MFR may indicate generalised coronary vascular dysfunction not apparent on semiquantitative imaging alone [34,56].

This pattern-based interpretation is critical, because relative perfusion imaging may appear normal despite balanced or global impairment in coronary flow.

#### 5.2.2. Thresholds and Standardisation Challenges

Although PET-derived myocardial flow reserve is a powerful integrative marker of coronary vascular health, its interpretation requires methodological caution.

Although thresholds vary across tracers and protocols, in clinical practice, an MFR value < 2.0 is generally considered abnormal and suggestive of impaired coronary vasodilator capacity. More severe reductions, particularly < 1.5 have been associated with higher-risk phenotypes and a greater likelihood of clinically relevant coronary vascular dysfunction. However, interpretation should consider haemodynamic conditions and methodological variability [49,52,57].

A further source of complexity is that MFR is a ratio and can therefore be reduced either because stress MBF is truly impaired or because resting MBF is disproportionately elevated. For this reason, interpretation should always consider resting haemodynamics, particularly heart rate, systolic blood pressure, and rate-pressure product, all of which can increase baseline myocardial blood flow and artifactually lower MFR despite near-normal hyperaemic flow [52,58,59]. Age and sex also influence physiological reference ranges: resting flow tends to increase with ageing, and sex-specific differences in MBF and MFR have been consistently documented [60]. Accordingly, isolated interpretation of a single global MFR value without clinical and haemodynamic context may lead to overcalling disease in some patients and under-recognising it in others [52].

These considerations explain why recent documents have strongly emphasised standardisation. Contemporary frameworks recommend structured reporting that includes, at minimum, global and regional rest MBF, stress MBF, and MFR, together with technical details relevant to reproducibility and quality control. This is particularly important in ANOCA/INOCA, where clinical interpretation often depends less on the binary presence of a perfusion defect and more on the integration of absolute flow values, regional distribution, haemodynamic context, and the likelihood of specific endotypes such as structural CMD, generalised atherosclerotic disease, or mixed physiology. Standardisation is therefore not merely a technical issue, but a prerequisite for broader clinical adoption and for meaningful comparison across centres [49,57,61].

#### 5.2.3. Prognostic Implications

In ANOCA/INOCA patients, a global reduction in MFR is a hallmark of structural coronary microvascular dysfunction and provides both diagnostic and prognostic information. The prognostic relevance of PET-derived flow impairment is one of its major strengths. Reduced MFR has been consistently associated with adverse cardiovascular outcomes, including major adverse cardiovascular events and all-cause mortality across a broad range of populations. Importantly, this prognostic signal is not confined to patients with obstructive epicardial coronary disease, reinforcing its clinical relevance [48]. Even when angiography does not show flow-limiting stenoses, impaired MFR identifies a subgroup with a higher-risk coronary vascular phenotype, consistent with underlying diffuse atherosclerosis, coronary microvascular dysfunction, or both [25,54].

In practical terms, PET-derived evidence of impaired flow reserve strengthens the case for mechanism-based management, more aggressive control of cardiometabolic risk factors, and closer follow-up in patients who might otherwise be falsely reassured by “normal” coronary anatomy [2,4,25].

#### 5.2.4. Limitations

Despite its considerable strengths, PET has important limitations that constrain its widespread implementation, since both availability and cost may represent a issue in some healthcare systems [48,52].

A second limitation is pathophysiological specificity. PET is excellent for quantifying abnormalities in coronary flow and vasodilator reserve, but it does not directly test vasomotor reactivity. Consequently, PET cannot by itself diagnose coronary spasm, nor can it reliably distinguish epicardial vasospasm from microvascular spasm. In patients in whom vasospastic angina is suspected, a normal or mildly abnormal PET study does not exclude dynamic vasomotor dysfunction, and invasive coronary function testing with vasoreactivity provocation remains necessary when endotype clarification is clinically important. PET should therefore be viewed as a powerful tool for identifying impaired flow regulation, but not as a complete substitute for invasive phenotyping when the diagnostic question specifically concerns coronary spasm [2,49,62].

### 5.3. Stress CMR: Quantitative Perfusion and Myocardial Phenotyping

#### 5.3.1. Quantitative Perfusion Mapping

Stress CMR has undergone a major methodological transition, evolving from a predominantly qualitative technique to a quantitative modality capable of measuring MBF and myocardial perfusion reserve (MPR) [51]. Recent developments in automated pixel-wise perfusion mapping allow absolute quantification of MBF (mL/min/g) at rest and during pharmacological stress, with improved reproducibility and reduced operator dependence, bridging the gap between non-invasive imaging and invasive physiology. These techniques enable detection of diffuse or balanced perfusion abnormalities that may be missed by visual assessment alone, particularly in patients with coronary microvascular dysfunction or extensive atherosclerosis [63,64]. Importantly, quantitative CMR-derived MBF shows good agreement with invasive physiological indices, including coronary flow reserve and microvascular resistance, supporting its role as a non-invasive surrogate of coronary vascular function [65]. In centres where PET is not available, quantitative stress CMR represents a powerful alternative for assessing coronary flow impairment, playing a central role in a multimodality, mechanism-based diagnostic strategy.

#### 5.3.2. Integrated Myocardial Characterisation

A major strength of CMR is its ability to combine perfusion assessment with tissue characterisation within a single examination. In addition to perfusion imaging, CMR provides late gadolinium enhancement (LGE), native T1 mapping, and extracellular volume (ECV) quantification. This multiparametric approach allows identification of alternative or concomitant myocardial pathologies, including prior infarction, myocarditis, infiltrative diseases, or cardiomyopathies, thereby reducing diagnostic misclassification in patients presenting with angina and non-obstructive coronary arteries and enabling more tailored management strategies [66,67,68].

#### 5.3.3. Diagnostic Performance and Incremental Value

Quantitative stress CMR provides incremental diagnostic value over qualitative perfusion imaging alone. Multicentre studies have demonstrated that absolute MBF and perfusion reserve outperform qualitative interpretation in detecting physiologically significant coronary artery disease, particularly in intermediate lesions and in patients with reduced ejection fraction, highlighting the added value of objective quantification [69,70,71]. In ANOCA/INOCA, this is particularly relevant, as quantitative CMR enables identification of microvascular dysfunction, often characterised by globally reduced perfusion without focal defects, and helps differentiate it from regional abnormalities suggestive of focal epicardial disease or mixed physiology [65,72].

#### 5.3.4. Limitations and Technical Considerations

Despite its strengths, quantitative stress CMR has several limitations. Image quality may be affected by arrhythmias, respiratory motion artefacts, breath-hold limitations, and contrast timing issues, all of which may reduce accuracy. Moreover, variability across vendors, acquisition protocols, and post-processing algorithms remains a significant challenge, limiting inter-centre reproducibility. Absolute MBF thresholds are also less well standardised compared with PET, and interpretation often requires integration with clinical and haemodynamic context [73].

### 5.4. PET Versus CMR: Complementary Rather than Competitive

PET and CMR should be considered complementary rather than competing modalities within a physiology-based diagnostic pathway. PET provides highly reproducible absolute quantification of myocardial blood flow and robust prognostic stratification, with well-established thresholds [55,56]. By contrast, CMR offers integrated myocardial phenotyping, combining quantitative perfusion with tissue characterisation in a single examination [66,67].

In clinical practice, the choice between PET and CMR is often driven by local availability and expertise. However, both modalities enable a quantitative, flow-based assessment of coronary physiology and should be viewed as complementary tools within a mechanism-oriented diagnostic framework. By identifying abnormalities in myocardial blood flow and flow reserve, they represent a critical step toward endotype-based diagnosis and guide subsequent invasive or targeted therapeutic strategies [2,4,49].

Synergistic use of PET and stress CMR provides comprehensive functional and structural assessment, enabling precise phenotypic characterisation of ANOCA/INOCA patients. Combining functional flow data from PET with tissue characterisation from CMR allows for targeted, individualised management strategies.

**Figure 1:**
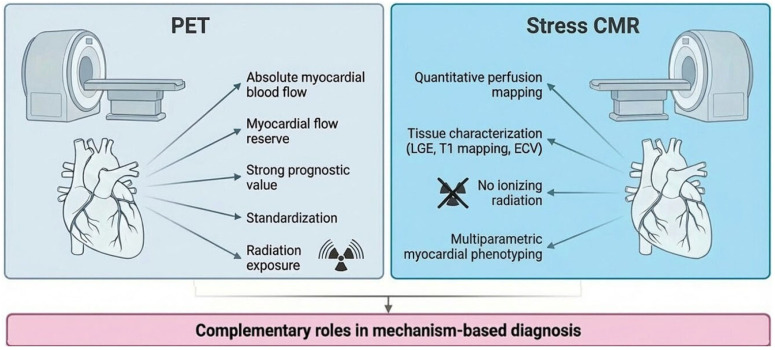
Complementary roles of PET and stress CMR in ANOCA/INOCA. Schematic comparison of PET and stress CMR in the non-invasive evaluation of coronary vascular dysfunction. PET provides highly reproducible absolute quantification of MBF and MFR, with robust prognostic value and established thresholds for risk stratification. In contrast, stress CMR enables integrated myocardial phenotyping by combining quantitative perfusion assessment with tissue characterisation, including LGE and parametric mapping. Rather than competing modalities, PET and CMR should be considered complementary tools within a mechanism-based diagnostic pathway, providing quantitative assessment of coronary flow and supporting endotype-oriented clinical decision-making. Abbreviations: PET, positron emission tomography; CMR, cardiovascular magnetic resonance; ANOCA, angina with non-obstructive coronary arteries; INOCA, ischaemia with non-obstructive coronary arteries; MBF, myocardial blood flow; MFR, myocardial flow reserve; LGE, late gadolinium enhancement.

The complementary roles of PET and stress CMR in the quantitative assessment of coronary physiology and endotype-oriented diagnosis are illustrated in Figure 1.

### 5.5. Bridge: From Anatomy to Physiology to Mechanism

In clinical practice, transition to invasive coronary function testing should be considered in patients with persistent angina, objective evidence of ischaemia, or recurrent symptoms despite non-invasive evaluation, particularly when non-invasive findings are inconclusive or discordant. Additional factors supporting referral include reduced myocardial flow reserve, unexplained symptom burden, or repeated healthcare utilisation in the absence of obstructive coronary artery disease.

These considerations highlight the limitations of an anatomy-centred diagnostic approach and support a stepwise transition toward physiology-guided evaluation. While CCTA provides an essential first-line assessment to exclude obstructive coronary artery disease and define atherosclerotic burden, it does not capture the functional integrity of the coronary circulation [1,4]. In contrast, non-invasive functional imaging with PET or quantitative stress CMR enables the assessment of myocardial blood flow and flow reserve, thereby identifying patients with impaired coronary vasodilator capacity despite non-obstructive coronary arteries [48,49,51].

However, although these techniques allow detection of flow impairment, they do not fully resolve the underlying mechanism. Reduced flow reserve may reflect different pathophysiological endotypes, including structural coronary microvascular dysfunction, extensive atherosclerosis, or vasomotor abnormalities, which cannot be reliably distinguished on the basis of non-invasive imaging alone [2,4].

In this context, invasive coronary function testing (ICFT) represents the critical next step, enabling comprehensive physiological characterisation of both the epicardial and microvascular compartments. This integrated pathway, from anatomical assessment to quantitative flow evaluation and ultimately to invasive mechanistic testing, reflects a paradigm shift from stenosis-centred to physiology- and endotype-oriented care in patients with ANOCA/INOCA.

**Figure 2:**
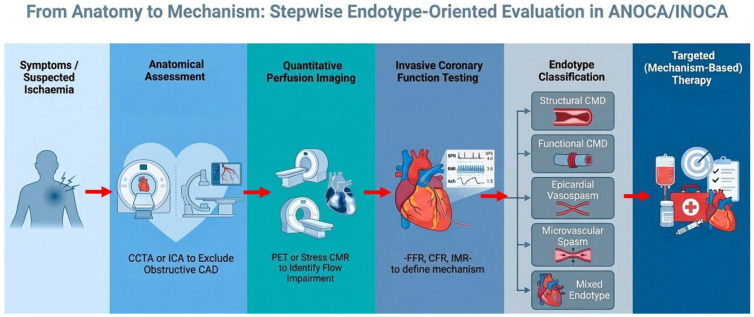
Mechanism-based diagnostic pathway in ANOCA/INOCA. Proposed stepwise diagnostic framework integrating anatomical imaging, quantitative perfusion assessment, and invasive coronary function testing in patients with angina and non-obstructive coronary arteries. The pathway illustrates the transition from exclusion of obstructive epicardial disease to identification of impaired coronary flow and final endotype definition, linking diagnostic findings to targeted therapy within a precision medicine approach. Abbreviations: ANOCA, angina and non-obstructive coronary arteries; CAD, coronary artery disease; CCTA, coronary computed tomography angiography; CFR, coronary flow reserve; CMD, coronary microvascular dysfunction; CMR, cardiovascular magnetic resonance; FFR, fractional flow reserve; ICA, invasive coronary angiography; INOCA, ischemia and non-obstructive coronary arteries; IMR, index of microcirculatory resistance; PET, positron emission tomography.

The complementary roles of anatomical imaging, quantitative perfusion assessment, and invasive physiology within an endotype-driven diagnostic pathway are summarised in Table 1 with a proposed stepwise diagnostic framework in Figure 2.

## 6. Invasive Coronary Function Testing: ESC-Aligned “Complete the Endotype” Strategy

### 6.1. Why ICFT Is Increasingly Central: Concept and Rationale

ICFT represents the most comprehensive diagnostic modality for mechanistic characterisation, enabling integrated assessment of epicardial physiology, coronary flow, microvascular resistance, and vasomotor function within a single procedural framework [1,4,8,74]. Unlike non-invasive imaging, which identifies the presence of flow impairment, ICFT allows direct interrogation of the underlying pathophysiological mechanisms [4,74].

ICFT is based on a structured multiparametric assessment combining indices of coronary flow, microvascular resistance, and vasomotor reactivity. Each component provides complementary information and contributes to endotype classification [2,4,62].

Contemporary evidence and recent guideline updates have progressively repositioned ICFT from a niche investigation to a central component of ANOCA/INOCA evaluation in selected patients, particularly those with persistent symptoms despite initial assessment or with discordant or inconclusive non-invasive findings [75]. Structured diagnostic pathways incorporating coronary function testing are increasingly recognised as essential for accurate diagnosis and targeted management of patients with ANOCA [2,4,42].

Contemporary guidelines and consensus documents increasingly support the use of ICFT in patients with persistent symptoms and non-obstructive coronary arteries, particularly when non-invasive testing is inconclusive or discordant [1,4,39,75].

### 6.2. Core Invasive Measurements: Epicardial Physiology, CFR, and Microvascular Resistance

A comprehensive invasive assessment requires the integration of multiple physiological domains rather than reliance on a single parameter. Epicardial physiology is typically assessed using FFR or non-hyperaemic pressure indices, with the primary aim of excluding flow-limiting stenoses.

Maximal hyperaemia is generally induced using adenosine, administered either intravenously or intracoronarily, to enable reliable assessment of pressure- and flow-derived indices, including FFR, CFR, and IMR. FFR ≤ 0.80 indicates haemodynamically significant epicardial disease [26,76,77,78].

However, the absence of a significant pressure gradient does not imply normal coronary physiology, as myocardial perfusion ultimately depends on flow rather than pressure alone [8,22].

Within this framework, CFR represents a key parameter, reflecting the capacity of the coronary circulation to augment blood flow in response to increased demand. CFR reflects the vasodilatory capacity of the coronary circulation and integrates both epicardial and microvascular contributions. A reduced CFR (<2.0–2.5, depending on methodology) indicates impaired coronary flow augmentation [20,74]. Nevertheless, CFR should be interpreted with caution, because it does not distinguish between epicardial stenosis and microvascular dysfunction as it integrates both components and therefore lacks specificity for isolating microvascular dysfunction [27,79]. Reduced CFR may reflect non-focal atherosclerosis, microvascular disease, or altered haemodynamic conditions, including elevated resting flow.

To overcome these limitations, indices of microvascular resistance, most commonly the IMR, provide a quantitative and relatively flow-independent measure of microvascular resistance, allowing specific assessment of the coronary microcirculation and a more direct assessment of the microcirculation by quantifying minimal resistance under hyperaemic conditions. Elevated IMR (≥25 units) is indicative of structural coronary microvascular dysfunction, offer greater specificity compared with flow-based indices alone and has been associated with adverse outcomes [77,78,80].

A critical principle in ICFT interpretation is therefore the integrated analysis of epicardial physiology, CFR, and microvascular resistance. CFR may be influenced by haemodynamic variability and non-focal epicardial disease, whereas resistance indices provide a more direct measure of microvascular integrity [22].

Only by combining these parameters can clinicians avoid misclassification and achieve an accurate definition of the underlying endotype.

### 6.3. Acetylcholine Provocation Testing: Epicardial vs. Microvascular Spasm

In addition to flow and resistance assessment, the evaluation of vasomotor function represents a fundamental component of ICFT. Intracoronary acetylcholine (ACh) testing is the reference standard for the diagnosis of coronary vasospastic disorders and is essential for distinguishing between epicardial vasospasm and microvascular spasm [5,81].

Pharmacological provocation testing with acetylcholine enables assessment of endothelial function and identification of epicardial and microvascular vasospasm. Epicardial vasospasm is defined by severe (>90%) transient and reversible coronary constriction associated with symptoms and/or ischaemic electrocardiographic changes, whereas microvascular spasm is diagnosed in the presence of symptoms and ECG changes without angiographic evidence of significant epicardial constriction and may be present in approximately 48% of patients [5,82].

Contemporary data have demonstrated a favourable safety profile of ACh testing when performed in experienced centres, and a positive response carries prognostic significance, identifying patients at increased risk of adverse cardiovascular events [81]. Accurate documentation of symptoms, ECG changes, angiographic findings, and response to nitrates is essential for correct interpretation.

From an implementation perspective, protocol standardisation and operator expertise are critical. Furthermore, ACh testing should be embedded within structured programmes with predefined safety protocols and post-test management pathways [40].

### 6.4. Integrated ICFT: Completing the Endotype

Based on ICFT findings, patients with ANOCA/INOCA can be stratified into distinct endotypes, each associated with specific pathophysiological mechanisms and therapeutic implications. This classification represents the cornerstone of mechanism-based management. The defining strength of ICFT lies not in individual measurements but in their integration within a unified physiological framework.

By combining epicardial pressure assessment, flow reserve, microvascular resistance, and vasoreactivity testing, ICFT enables classification into clinically actionable endotypes, including structural and functional coronary microvascular dysfunction, epicardial vasospasm, microvascular spasm, and mixed phenotypes [4,75].

Despite its diagnostic value, ICFT is not without limitations. Both false-negative and false-positive results may occur, particularly in the context of variable haemodynamic conditions, suboptimal hyperaemia, or incomplete vasoreactivity testing. For example, inadequate pharmacological provocation may underestimate vasospastic disorders, while microvascular indices may be influenced by resting haemodynamics or technical factors.

Furthermore, the dynamic nature of vasomotor disorders may lead to intermittent findings, potentially contributing to diagnostic variability. These considerations highlight the importance of standardised protocols, operator expertise, and integrated interpretation of all physiological parameters within the clinical context [15,44,62,75].

Discordant physiological patterns, such as preserved FFR with reduced CFR or normal resistance indices with abnormal vasoreactivity, should be interpreted as expressions of the multidimensional nature of coronary dysfunction rather than inconsistencies [22,27].

**Figure 3:**
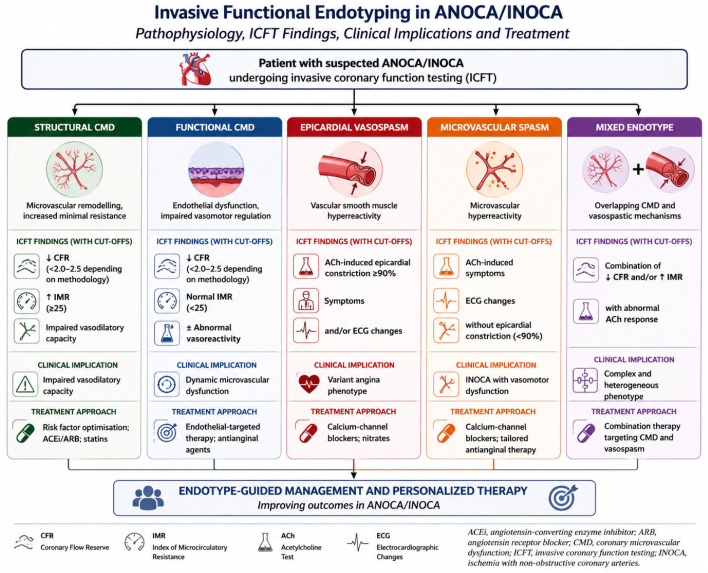
Core invasive coronary function testing parameters and endotype classification. Overview of the principal ANOCA/INOCA endotypes as defined by invasive coronary function testing, including underlying pathophysiology, characteristic physiological findings, clinical implications, and corresponding treatment strategies. The figure illustrates how integration of coronary flow reserve, microvascular resistance indices, and vasoreactivity testing enables mechanism-based classification. Cut-off values may vary according to methodology and haemodynamic conditions and should always be interpreted within the clinical context.

Key invasive physiological measurements, their diagnostic interpretation, and principal limitations are showed in Figure 3.

### 6.5. Evidence of Clinical Utility: CorMicA and Stratified Therapy

The clinical relevance of ICFT is supported by prospective evidence demonstrating that a stratified, mechanism-based approach improves patient outcomes. The CorMicA trial demonstrated that an interventional diagnostic strategy incorporating coronary function testing, followed by stratified therapy based on identified endotypes, resulted in significant improvements in angina symptoms and quality of life compared with standard care [42]. Importantly, these benefits were sustained at one-year follow-up, reinforcing the durability of this approach [43].

More recent real-world data further confirm the feasibility, safety, and high diagnostic yield of comprehensive coronary function testing, with a large proportion of ANOCA patients demonstrating identifiable endotypes when systematically evaluated [75].

These findings confirm that coronary vascular dysfunction is not a benign condition but a clinically actionable disease process, in which accurate mechanistic diagnosis directly translates into more effective, individualised treatment strategies. Collectively, these data support a paradigm in which ICFT is not merely a diagnostic adjunct but a central tool for implementing precision medicine in ANOCA/INOCA [4,8].

## 7. Linking Endotype to Therapy

### 7.1. From Mechanism to Management: A Paradigm Shift

The management of ANOCA/INOCA has historically been limited by a mismatch between diagnosis and treatment, with therapeutic strategies often applied empirically in the absence of a clearly defined mechanism. The recognition of coronary vascular dysfunction as the principal substrate underlying these syndromes has enabled a transition toward a mechanism-based paradigm, in which treatment is guided by the identification of specific coronary endotypes [15].

Within this framework, the goal of diagnostic evaluation extends beyond confirming the presence of ischaemia to defining the dominant pathophysiological process driving symptoms. This approach enables clinicians to move beyond non-specific antianginal therapy and implement targeted, endotype-oriented strategies aligned with the underlying biology of disease [2,4].

Importantly, contemporary physiological insights highlight that discordance between anatomical and functional indices is not uncommon and reflects complex coronary pathophysiology rather than measurement error, reinforcing the need for integrated assessment of coronary flow and resistance [75].

Invasive physiological assessment, including indices such as coronary flow reserve and index of microcirculatory resistance, provides mechanistic insight into this phenotype and may guide therapeutic decisions [77,78].

### 7.2. Structural Coronary Microvascular Dysfunction: A Systemic Cardiometabolic Target

Structural CMD is characterised by impaired vasodilatory capacity and increased microvascular resistance, reflecting fixed alterations of the coronary microcirculation [26,77].

Accordingly, therapeutic strategies should primarily target systemic determinants of microvascular dysfunction rather than isolated coronary vasodilation. Aggressive optimisation of cardiovascular risk factors represents the cornerstone of management, including lipid-lowering therapy, blood pressure and glycaemic control, and lifestyle interventions [25,52]. Pharmacological strategies such as statins and renin–angiotensin system inhibitors may exert beneficial effects beyond risk reduction, including improvement in endothelial function and microvascular remodelling [52,82,83].

From a symptomatic perspective, beta-blockers are often considered first-line therapy, particularly when elevated heart rate and myocardial oxygen demand contribute to symptom burden. In selected patients, ivabradine may also be considered as a heart rate-lowering strategy, especially when reduction in myocardial oxygen demand and prolongation of diastolic perfusion time are desirable, although evidence in ANOCA/INOCA remains less robust than for broader cardiometabolic optimisation [41,82,83].

Ranolazine may be considered in patients with persistent angina despite conventional therapy. By inhibiting the late sodium current, it reduces intracellular calcium overload, improves diastolic relaxation, and may enhance subendocardial perfusion. This mechanism may be particularly relevant in patients with diabetes, in whom metabolic derangements, impaired myocardial relaxation, and microvascular dysfunction frequently coexist, potentially making ranolazine especially attractive in this phenotype, although therapeutic response remains heterogeneous [41,53,82,83].

These therapeutic considerations are supported by physiological studies demonstrating the association between microvascular dysfunction, impaired perfusion during stress, and symptom burden [79]. Overall, structural CMD should be conceptualised as a systemic cardiometabolic disease of the microcirculation requiring comprehensive risk factor modification rather than purely antianginal therapy.

### 7.3. Functional CMD: Targeting Endothelial Dysfunction

Functional (endothelial-dependent) CMD represents a dynamic phenotype characterised by impaired endothelial-mediated vasodilation and abnormal regulation of vascular tone [13,28]. Therapeutic strategies should aim to restore endothelial function and improve vasomotor regulation.

Optimisation of cardiovascular risk factors remains essential, with particular emphasis on interventions known to improve endothelial function, including renin-angiotensin system inhibition, statins, and lifestyle modification [2,52,83]. However, treatment response is often heterogeneous, reflecting the complex interplay between endothelial dysfunction, microvascular resistance, and vasomotor abnormalities [13,15].

In selected symptomatic patients, antianginal agents such as ranolazine or ivabradine may be considered on an individual basis, particularly when persistent microvascular angina, elevated heart rate, or impaired diastolic perfusion remain clinically relevant, although supportive evidence is less consistent than in obstructive chronic coronary syndromes [41,82,83].

Importantly, functional CMD frequently overlaps with vasospastic disorders, and treatment strategies may require incorporation of vasodilator agents when vasomotor dysfunction is present [4,15].

### 7.4. Vasospastic Angina and Microvascular Spasm: Suppressing Abnormal Vasoconstriction

Vasospastic angina is characterised by transient coronary vasoconstriction driven by vascular smooth muscle hyperreactivity and endothelial dysfunction. In contrast to CMD, where impaired vasodilation predominates, the primary therapeutic target is suppression of abnormal vasoconstrictive activity [29,31,39,40].

Calcium-channel blockers represent first-line therapy and are highly effective in preventing vasospastic episodes, while long-acting nitrates may be used as adjunctive therapy in patients with persistent symptoms [39,40,83]. Avoidance of triggers, including smoking, sympathomimetic agents, and emotional stress, is essential. Non-selective beta-blockers may exacerbate vasospasm and should be used with caution [29,31,39,40].

Microvascular spasm shares similar mechanisms but occurs at the level of the microcirculation and is not detectable on angiography. Its diagnosis relies on invasive vasoreactivity testing, including acetylcholine provocation, which has demonstrated both diagnostic and prognostic value [81].

Therapeutic management is broadly aligned with that of epicardial vasospasm, although treatment response is often more variable and requires individualization [61,82,83].

### 7.5. Mixed Endotypes: The Rule Rather than the Exception

Contemporary evidence indicates that mixed endotypes are highly prevalent in ANOCA/INOCA, with overlapping features of CMD and vasospastic disorders observed in a substantial proportion of patients. Mixed endotypes may be present in up to 40–60% of ANOCA patients undergoing comprehensive physiological assessment [11,33,38]. This overlap reflects the complex interplay between microvascular dysfunction and vasomotor abnormalities and may explain discordant findings across diagnostic modalities [23,24]. In clinical practice, combination therapy is frequently required. Calcium-channel blockers may be used to control vasospasm, while concurrent optimisation of cardiometabolic factors and microvascular-directed therapies address underlying CMD [28,52,82].

The identification of mixed endotypes represents a major advantage of comprehensive physiological assessment, enabling tailored therapy and reducing reliance on empiric treatment strategies.

### 7.6. Evidence Supporting Stratified Therapy

The clinical relevance of endotype-based management is supported by interventional evidence. In the CorMicA trial, a diagnostic strategy incorporating invasive coronary function testing followed by stratified therapy resulted in significant improvements in angina severity and quality of life [42,43]. Mechanistic studies further support the link between coronary microvascular dysfunction, impaired stress perfusion, and clinical outcomes, reinforcing the rationale for targeted treatment strategies [79,80]. Collectively, these findings support the integration of endotype-guided therapy into routine clinical practice.

The main therapeutic implications of endotype classification, including first-line and adjunctive treatment options, are summarised in Table 2.

### 7.7. Toward Precision Medicine in ANOCA/INOCA

Taken together, these observations support a paradigm in which ANOCA/INOCA is no longer considered a homogeneous entity but rather a spectrum of pathophysiological processes requiring tailored management. The integration of multimodality imaging and invasive coronary function testing enables identification of clinically actionable endotypes, providing the foundation for precision medicine in coronary vascular dysfunction.

In line with recent national consensus efforts, increasing attention is being directed towards mechanism-based diagnostic pathways in ANOCA/INOCA, incorporating both invasive functional assessment and advanced non-invasive imaging [84].

Future research should focus on refining endotype classification, improving non-invasive surrogates of invasive physiology, and evaluating the impact of mechanism-based therapies on long-term cardiovascular outcomes.

**Figure 4:**
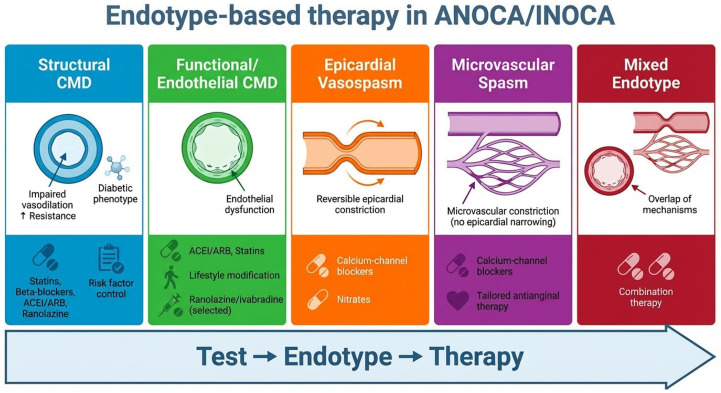
Endotype-based therapeutic approach in ANOCA/INOCA. Schematic representation of the principal ANOCA/INOCA endotypes and their corresponding mechanism-based treatment strategies. Structural and functional coronary microvascular dysfunction, epicardial vasospasm, microvascular spasm, and mixed phenotypes are linked to targeted therapies, emphasising the transition from empirical treatment to precision, endotype-driven management. Abbreviations: ACEi, angiotensin-converting enzyme inhibitors; ANOCA, angina with non-obstructive coronary arteries; ARB, angiotensin receptor blockers; CCB, calcium-channel blockers; CMD, coronary microvascular dysfunction; INOCA, ischaemia with non-obstructive coronary arteries.

A summary of endotype-specific therapeutic strategies within a mechanism-based framework is provided in Figure 4.

## 8. Conclusions

### 8.1. Practical Implementation and Barriers

Despite its conceptual appeal, a mechanism-based ANOCA/INOCA pathway must be adapted to real-world resource availability.

Quantitative PET, advanced stress CMR, and comprehensive ICFT are not uniformly accessible, and implementation may be limited by cost, local expertise, radiotracer availability, catheterisation laboratory workflow, and the need for dedicated training in coronary vasomotor testing. Therefore, the proposed paradigm should not be interpreted as requiring all tests in every patient, but rather as a stepwise strategy guided by symptoms, pre-test probability, previous test results, and local resources.

In centres with limited access to PET or ICFT, a pragmatic pathway may begin with careful clinical phenotyping, exclusion of obstructive coronary artery disease by CCTA or invasive angiography, optimisation of cardiovascular risk factors, and use of available functional imaging such as stress CMR, stress echocardiography, or SPECT when quantitative techniques are unavailable.

Patients with persistent angina, objective ischaemia, recurrent healthcare utilisation, or discordant non-invasive findings should be considered for referral to centres with expertise in quantitative perfusion imaging and invasive coronary function testing. From a health-system perspective, broader adoption will require standardised referral pathways, structured reporting, operator training, and integration of ICFT into catheterisation laboratory protocols.

Although cost-effectiveness data remain limited, improved diagnostic certainty, reduced repeated testing, and more targeted therapy may offset the upfront costs of specialised testing in selected patients. Future implementation studies should define which patients derive the greatest clinical and economic benefit from advanced endotype-guided evaluation.

### 8.2. Future Perspectives

Future progress in the management of ANOCA/INOCA will depend on the integration of quantitative physiology into routine clinical practice and the transition from descriptive diagnosis to mechanism-based care. A key priority is the broader implementation of quantitative perfusion imaging, including both PET and stress CMR, supported by harmonised acquisition protocols and standardised reporting frameworks. Although PET-derived myocardial blood flow and flow reserve currently represent the non-invasive reference standard for coronary physiology, advances in quantitative CMR, including automated pixel-wise perfusion mapping, are expanding access to flow-based assessment and reducing reliance on operator-dependent interpretation [62,72].

Standardisation remains a critical challenge. Variability in thresholds for myocardial blood flow and flow reserve across tracers, imaging platforms, and software pipelines limits comparability and clinical adoption. The development of validated, population-adjusted reference values and consensus reporting standards will be essential to ensure reproducibility and facilitate integration into clinical decision-making. In parallel, the incorporation of artificial intelligence–assisted analysis may improve efficiency, reduce interobserver variability, and enable wider dissemination of quantitative imaging beyond specialised centres.

Equally important is the development of integrated clinical pathways embedding invasive coronary function testing within catheterisation laboratory programmes. Implementation studies aligned with ESC recommendations emphasise the need for dedicated training, standardised protocols, and structured reporting systems to ensure safe and effective delivery of ICFT in routine practice [18].

Such programmes are essential to translate guideline recommendations into real-world care and to reduce the persistent diagnostic gap in ANOCA/INOCA. From a research perspective, the next frontier lies in endotype-guided clinical trials. While current evidence supports the symptomatic benefit of stratified therapy, further studies are needed to evaluate the impact of mechanism-based treatment on long-term outcomes, including major adverse cardiovascular events and healthcare utilisation [25,36,42,43,80].

In addition, prospective studies integrating non-invasive imaging and invasive physiology will be crucial to refine diagnostic algorithms and validate non-invasive surrogates of coronary endotypes. In conclusion, ANOCA/INOCA represents a paradigm in which traditional stenosis-centred approaches are insufficient to explain symptoms, guide therapy, or predict outcomes.

Multimodality evaluation should therefore be reframed as a structured endotyping strategy rather than sequential test accumulation. An ESC-aligned approach integrates anatomical assessment to exclude obstructive coronary artery disease, quantitative perfusion imaging to identify diffuse flow abnormalities, and invasive coronary function testing to define specific endotypes.

Evidence demonstrates that an invasive diagnostic strategy linked to stratified therapy improves symptoms and quality of life, highlighting the clinical value of mechanism-based care. By linking diagnostic findings to targeted treatment, a test-to-endotype-to-therapy framework has the potential to close the longstanding gap between diagnosis and management in patients with ischaemia beyond stenosis.

## Figures and Tables

**Table 1:** Endotype-driven diagnostic framework in ANOCA/INOCA. Summary of the main diagnostic modalities used in the evaluation of patients with ANOCA/INOCA, including their key outputs, associated endotype signals, clinical role, limitations, and therapeutic implications. The table highlights the complementary contribution of anatomical imaging, quantitative perfusion assessment, and invasive coronary function testing within a mechanism-based diagnostic strategy.

Modality/Test	Key Outputs	Endotype Signal	Clinical Role	Limitations	Therapeutic Implications
**CCTA**	Stenosis severity; plaque burden and phenotype	Non-obstructive plaque → atherosclerotic substrate; normal CCTA does not exclude CMD or vasospasm	Anatomical gatekeeper; selection for functional testing	Heavy calcification; motion artefacts	Intensify preventive therapy; proceed to functional testing if symptoms persist
**Stress PET (MBF/MFR)**	Rest and stress MBF; global and regional MFR	Global reduction in MFR/MBF → structural CMD or diffuse coronary dysfunction	First-/second-line functional assessment	Haemodynamic variability; tracer and software differences; limited detection of spasm	Supports CMD diagnosis; risk stratification; guides cardiometabolic optimisation
**Stress CMR (quantitative)**	Stress MBF/MPR; regional perfusion; LGE; T1/ECV	Extensive perfusion impairment → CMD; tissue abnormalities may suggest alternative myocardial pathology	Alternative to PET; integrated “one-stop” evaluation	Arrhythmias; motion artefacts; variability in analysis pipelines	Guides CMD management; avoids misclassification; identifies alternative myocardial pathology
**ICA + FFR/iFR**	Epicardial physiology	Flow-limiting epicardial disease	Assessment of obstructive CAD prior to ICFT; excludes focal flow-limiting epicardial disease	Does not directly assess coronary blood flow or microvascular dysfunction; non-focal atherosclerotic disease may affect interpretation	Guides revascularisation decisions; a normal FFR/iFR does not exclude coronary vascular dysfunction
**CFR (wire-based)**	Coronary flow reserve	Reduced CFR suggests CMD but lacks specificity	Component of ICFT	Influenced by haemodynamics and epicardial disease	Requires integration with resistance indices and vasoreactivity testing
**Microvascular resistance (IMR)**	Microvascular resistance index	Elevated resistance → structural CMD	Core component of microvascular assessment	Technique-dependent	Supports CMD-targeted therapy; avoids inappropriate vasodilator-only strategies
**Acetylcholine provocation**	Vasomotor response; symptoms; ECG changes	Epicardial vs. microvascular spasm; mixed phenotypes	Diagnosis of vasospastic disorders	Requires expertise; protocol variability	Calcium-channel blockers first-line; nitrates and trigger modification
**Integrated ICFT**	Comprehensive physiological assessment	CMD, vasospasm, or mixed endotypes	ESC-recommended in persistent symptoms	Requires dedicated infrastructure	Enables stratified therapy; improves symptoms and quality of life

Abbreviations: ANOCA, angina and non-obstructive coronary arteries; CAD, coronary artery disease; CCTA, coronary computed tomography angiography; CFR, coronary flow reserve; CMD, coronary microvascular dysfunction; CMR, cardiovascular magnetic resonance; ECV, extracellular volume; FFR, fractional flow reserve; ICA, invasive coronary angiography; ICFT, invasive coronary function testing; iFR, Instantaneous Wave-Free Ratio; INOCA, ischemia and non-obstructive coronary arteries; IMR, index of microcirculatory resistance; LGE, late gadolinium enhancement; MBF, myocardial blood flow; MFR, myocardial flow reserve; PET, positron emission tomography.

**Table 2:** Endotype-specific therapeutic strategies in ANOCA/INOCA. Overview of mechanism-based therapeutic approaches according to coronary endotype, including key diagnostic features, underlying pathophysiology, first-line treatments, adjunctive therapies, and clinical considerations. The table reflects a test-to-endotype-to-therapy paradigm, emphasising the importance of aligning treatment with the dominant pathophysiological mechanism. Therapeutic responses may vary, particularly in overlapping or mixed endotypes, requiring individualised management strategies.

Endotype	Pathophysiological Target	First-Line Therapy	Mechanism-Based Pharmacological Strategies	Clinical Considerations
**Structural CMD**	Increased microvascular resistance, impaired vasodilatory capacity	Risk factor modification + β-blockers	ACEi/ARBs and statins to improve endothelial function and microvascular remodelling; β-blockers to reduce myocardial oxygen demand; consider ranolazine or ivabradine in selected patients	Disease-modifying therapy is central; focus on cardiometabolic optimisation rather than pure vasodilation
**Functional (endothelium-dependent) CMD**	Endothelial dysfunction, impaired NO-mediated vasodilation	ACEi/ARBs + statins	Therapies targeting endothelial function (ACEi, statins); consider CCBs if vasomotor component present; ranolazine may improve perfusion in selected patients	Overlap with vasospasm is common; treatment response may be heterogeneous
**Endothelium-independent CMD**	Impaired microvascular vasodilatory response (adenosine pathway)	β-blockers (particularly when increased myocardial oxygen demand predominates)	β-blockers to reduce demand; consider adjunctive agents (ranolazine, ivabradine, nicorandil) in persistent symptoms	Requires differentiation from endothelial dysfunction; often identified by reduced CFR/IMR abnormalities
**Epicardial VSA**	Hyperreactivity of vascular smooth muscle, transient epicardial spasm	CCBs	Dihydropyridine or non-dihydropyridine CCBs; long-acting nitrates as second-line; nicorandil in refractory cases	Avoid non-selective β-blockers; trigger control (e.g., smoking) is essential
**Microvascular spasm**	Microvascular vasoconstriction (ACh-induced)	CCBs	Similar to vasospastic angina; consider nitrates and nicorandil; potential role for ranolazine or Rho-kinase inhibitors (investigational)	Diagnosis requires invasive testing; response often variable
**Mixed endotypes**	Combined CMD + vasospasm	CCBs + risk factor control	Combination therapy targeting both vasodilation and microvascular dysfunction (CCBs + ACEi/ARB + statins ± antianginals)	Present in up to 40–60% of patients; requires individualised therapy

Abbreviations: CMD, coronary microvascular dysfunction; VSA, vasospastic angina; ACEi, angiotensin-converting enzyme inhibitors; ARBs, angiotensin receptor blockers; CCBs, calcium-channel blockers; NO, nitric oxide; CFR, coronary flow reserve; IMR, index of microcirculatory resistance; ACh, acetylcholine.

## Data Availability

No new data were created or analyzed in this study.

## Abbreviations

The following abbreviations are used in this manuscript:

ACh	acetylcholine
ACEi	angiotensin-converting enzyme inhibitors
ANOCA	angina with non-obstructive coronary arteries
ARB	angiotensin receptor blockers
CAD	coronary artery disease
CCB	calcium-channel blockers
CCS	chronic coronary syndromes
CCTA	coronary computed tomography angiography
CFR	coronary flow reserve
CMD	coronary microvascular dysfunction
CMR	cardiovascular magnetic resonance
COVADIS	Coronary Vasomotion Disorders International Study Group
ECG	electrocardiogram
ECV	extracellular volume
ESC	European Society of Cardiology
FFR	fractional flow reserve
FFR-CT	computed tomography-derived fractional flow reserve
ICA	invasive coronary angiography
ICFT	invasive coronary function testing
iFR	instantaneous wave-free ratio
IMR	index of microcirculatory resistance
INOCA	ischaemia with non-obstructive coronary arteries
LGE	late gadolinium enhancement
MBF	myocardial blood flow
MFR	myocardial flow reserve
MPR	myocardial perfusion reserve
NO	nitric oxide
PET	positron emission tomography
VSA	vasospastic angina
